# Oxidative Stress in Canine Diseases: A Comprehensive Review

**DOI:** 10.3390/antiox13111396

**Published:** 2024-11-15

**Authors:** Perez-Montero Blanca, Fermín-Rodriguez María Luisa, Miró Guadalupe, Cruz-Lopez Fátima

**Affiliations:** 1 Clinical Pathology Service, Veterinary Teaching Hospital, Complutense University, 28040 Madrid, Spain; mfermin@ucm.es; 2 Animal Medicine and Surgery Department, Faculty of Veterinary Medicine, Complutense University, 28040 Madrid, Spain; 3 Animal Health Department, Faculty of Veterinary Medicine, Complutense University, 28040 Madrid, Spain; gmiro@ucm.es; 4 VISAVET Health Surveillance Centre, Complutense University, 28040 Madrid, Spain; fatimacr@ucm.es

**Keywords:** oxidation, antioxidants, redox, dog, free radicals, veterinary medicine

## Abstract

Oxidative stress (OS), defined as a disruption in redox balance favoring oxidants, has emerged as a major contributor to numerous diseases in human and veterinary medicine. While several reviews have explored the implication of OS in human pathology, an exhaustive review of the canine species is lacking. This comprehensive review aims to summarize the existing literature on the role of OS in canine diseases, highlighting its potentially detrimental effect on various organs and systems. Some inconsistencies among studies exist, likely due to varying biomarkers and sample types. However, there is substantial evidence supporting the involvement of OS in the development or progression of numerous canine disorders, such as cardiovascular, oncologic, endocrine, gastrointestinal, hematologic, renal, neurologic, infectious, and parasitic diseases, among others. Additionally, this review discusses the efficacy of antioxidant and pro-oxidant therapeutic agents for these conditions. Dietary interventions to counteract OS in dogs have gained significant attention in recent years, although further research on the topic is needed. This review aims to serve as a foundational resource for future investigations in this promising field.

## 1. Introduction

Reduction-oxidation (redox) reactions are central mechanisms of life in biological systems [1]. The concept of “oxidative stress” (OS) arose in 1985, describing a potentially harmful imbalance between the production of oxidants and the organism’s antioxidant defenses, favoring the oxidants [2]. Since then, the knowledge of redox biology has undergone significant advancements and the concept has been redefined to account for the broad implications of redox homeostasis [1,3,4,5]. Pro-oxidant agents include a wide variety of molecules, some of which are free radicals and others are non-radicals, collectively referred to as ‘reactive species’, such as reactive oxygen species (ROS), reactive nitrogen species (RNS), and others [6]. These species originate from endogenous sources, such as normal cellular metabolism, inflammation, or immune cell activation, as well as from exogenous sources, including exposure to pollutants, chemicals, or radiation [4,7,8]. Several metals and metalloproteins, such as iron, copper, chromium, and cobalt, also contribute to the generation of reactive species, primarily through Fenton-like reactions [9,10].

When maintained at controlled concentrations, reactive species play a crucial role in various physiological processes, including cellular signaling, phagocytosis, and regulation of vascular tone [8,11,12]. Nonetheless, elevated levels of reactive species and their by-products can cause severe damage to biomolecules and contribute to the pathogenesis of numerous diseases [1,3,5].

Given the variety of compounds and pathways implicated in redox regulation, numerous methods have been developed to evaluate OS, leading to the identification of multiple measurable biomarkers. The direct measurement of reactive species (ROS, RNS) and reactive oxygen metabolites (d-ROMs) can be challenging due to their very short half-life and the requirement for expensive equipment. Therefore, a more practical approach involves measuring quantifiable products of oxidative damage to biomolecules, such as lipids, proteins, and DNA [13,14,15]. The most frequently measured biomarkers of lipid peroxidation, such as the oxidation products of polyunsaturated fatty acids (PUFAs), are malondialdehyde (MDA), 4-hydroxy-2nonenal (4-HNE), Isoprostanes (IsoP), and acrolein [15,16]. Reactive species can also lead to DNA modifications in several ways, resulting in DNA-oxidation biomarkers such as 8-hydroxy-2′-deoxyguanosine (8-OHdG), one of the most extensively studied [7,17,18]. The oxidation of proteins can be measured as advanced oxidation protein products (AOPPs) and protein carbonyls (PCs) [19]. Another approach to evaluating OS is assessing antioxidant defenses, which include non-enzymatic agents (e.g., vitamins, metals (selenium, zinc), thiol groups, and reduced glutathione (GSH)) and numerous antioxidant enzymes, such as glutathione peroxidase (GPX), superoxide dismutase (SOD), or catalase (CAT) [7,15,19,20]. Additionally, several widely used indexes reflect the overall antioxidant capacity of a sample, such as the 2,2′-azinobis(3-ethylbenzthiazolin-6-sulfonic acid) (ABTS) test, also known as the Total Antioxidant Status (TAS) assay; the Cupric-Reducing Antioxidant Power (CUPRAC) test; and the Ferric-Reducing Antioxidant Power (FRAP) assay [19,21,22,23]. Table 1 summarizes the most common OS biomarkers used in the literature. Further information on this topic can be found in the reviews by Tejchman et al. [15], Frijhoff et al. [18], Sánchez-Rodríguez et al. [19], Dalle-Donne et al. [13], and Sies et al. [1].

**Figure 1:**
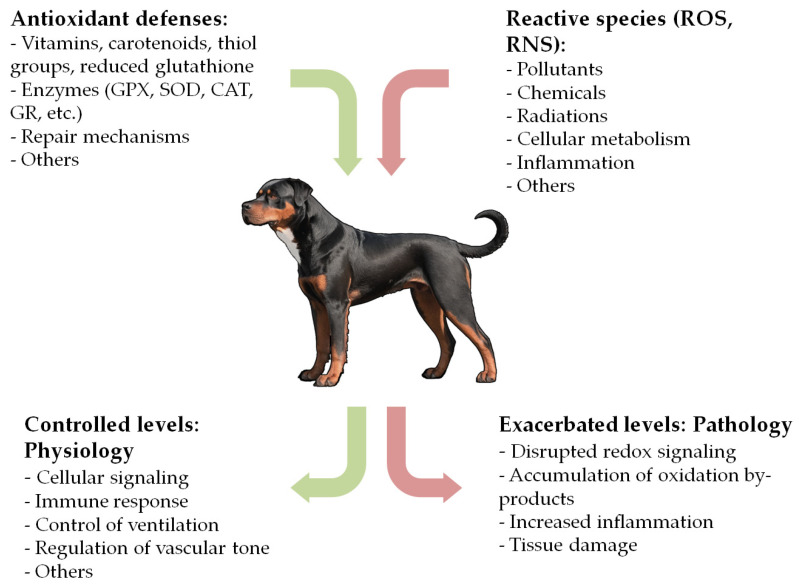
Simplified mechanisms of oxidative stress.

OS has been implicated in the development and progression of numerous diseases in humans. While the exact mechanisms in some cases remain unclear, a triad of OS, inflammation, and functional impairment appears to be involved in the pathogenesis of many clinical conditions [1,3,8,15,24]. In recent years, this field has gained growing interest in veterinary medicine, with mounting evidence suggesting a relevant role of OS in the pathogenesis of many canine diseases (Figure 1). Redox homeostasis in dogs also appears to be influenced by factors such as psychogenic stress, housing, and exercise, though this aspect remains unclear [23,25,26,27,28]. While numerous reviews exist in human medicine, to the best of our knowledge, no comprehensive review has been conducted for the canine species. To fill this gap, we performed an extensive literature search in the PubMed database, focusing on original research articles, experimental studies, clinical trials, and reviews related to the association between OS and canine diseases. Search terms included ‘oxidative stress’, ‘oxidation’, ‘antioxidant’, ‘redox’, ‘dog’, ‘canine’, ‘disease’, ‘disorder’, and ‘pathology’. Each selected article was carefully reviewed, and the final version of the manuscript includes relevant literature published up to November 2024. The main sections of this manuscript examine the role of OS in cardiovascular, respiratory, oncologic, gastrointestinal, hepatobiliary, endocrine, hematologic, infectious, parasitic, neurologic, renal, dermatologic, ophthalmologic, orthopedic, reproductive, dental, and other canine diseases (Table 2). This review aims to consolidate current evidence and provide a comprehensive overview of this expanding field.

## 2. OS in Canine Diseases

### 2.1. Cardiovascular, Respiratory, and Related Diseases

Cardiovascular diseases are likely among the most extensively studied OS-related pathologies in both human and canine medicine. Cardiac tissue has several sources of reactive species, primarily the mitochondrial electron transport chain, followed by various enzymatic sources such as xanthine oxidase, NADPH oxidase (NOX), and nitric oxide (NO) synthase, among others [12,29,30]. At moderate levels, reactive species play physiological roles, such as regulating vascular tone and signaling cascades in cardiac myocytes. However, increased ROS formation damages subcellular organelles, leading to myocyte contractile dysfunction, loss of functional myocardium, and a decrease in cardiac output. As a result, OS has been linked to various pathological conditions in humans, including hypertension, atherosclerosis, myocardial infarction, ischemia/reperfusion, and heart failure [8,11,12,29,31].

Consistent evidence supports that OS is also present in canine cardiovascular pathology. Several circulating biomarkers of oxidation, mainly lipid peroxidation markers such as MDA and IsoP, as well as antioxidant defense markers, including vitamins, enzymes, and total antioxidant capacity indexes, have been studied in dogs with myxomatous mitral valve disease (MMVD) and dilated cardiomyopathy (DCM) [32,33,34,35,36,37,38,39,40,41,42]. While some studies show discrepancies in results, most report significant differences in OS biomarkers between dogs with MMVD or DCM and healthy controls, suggesting increased oxidative assault and a decline in the efficacy of the antioxidant forces [32,33,34,35,36,37,38,40,41]. Additionally, some studies have shown significant correlations between OS parameters and cytokines, natriuretic peptides, other inflammatory markers, or echocardiographic measurements in dogs with heart failure. This suggests a combined effect of oxidative and inflammatory processes in these patients [38,40,42]. A few studies have failed to show such OS changes in dogs with cardiac conditions, possibly due to the specific biomarkers studied, non-linear changes in oxidation across stages of MMVD or DCM, or potential antioxidant effects of some therapeutic agents such as benazepril or sildenafil, which have yet to be conclusively studied [32,39,42].

To address potential complications arising from OS in dogs with heart disease, several studies have evaluated the efficacy of nutrients and antioxidant administration, especially in dogs with MMVD. Supplementation with specific lipids (omega-3 PUFAs or medium-chain triglycerides) and other compounds (e.g., magnesium, methionine, or lysine) seems to provide benefits by reducing mitochondrial ROS production and supporting other metabolic functions [43]. Boosting antioxidant defenses through the administration of vitamins, taurine, melatonin, and atorvastatin has also shown cardioprotective effects by attenuating OS in dogs with heart disease [43,44,45]. In contrast, Coenzyme Q10 has not demonstrated similar benefits [46].

Additionally, canine models have been extensively used to study induced atrial fibrillation [47,48,49,50,51,52], cardiac arrest [53,54], and heart failure [55,56]. Recent studies have demonstrated increased levels of oxidation markers (ROS, 8-OHdG) and decreased antioxidant enzymes (GPX, SOD) in the blood and cardiac tissue of dogs with induced atrial fibrillation, suggesting an important role of OS in promoting atrial tissue fibrosis, conduction disturbances, and therefore, atrial arrhythmias [47,48,49,50,51,52]. These negative effects have been shown to be attenuated by antihypertensive [52] and antidiabetic [47,49] drugs, among others [48,50,51].

Some cardiorespiratory diseases in dogs, such as tracheal collapse, Brachycephalic Obstructive Airway Syndrome, and exposure to pollutants, have also been linked to OS, possibly associated with inflammatory stages or recurrent hypoxia/reoxygenation events [57,58,59,60,61]. Oxidation markers (MDAs) have been reported to decrease in dogs with tracheal collapse receiving acupuncture and fatty acid supplementation [57,61], and increased antioxidant enzymes (SODs) have been found in dogs with Brachycephalic Obstructive Airway Syndrome after corrective surgery [58]. Conversely, one study did not detect significant differences in lipid (MDA) and protein oxidation (PC) markers in a canine model of hypoxia-induced neurogenic pulmonary edema [62]. Exposure to chromium and petrol generator exhaust fumes, simulating highly polluted environments, has also been linked to increased oxidation (MDA, ROS) and inflammatory biomarkers, along with a decrease in certain antioxidant enzymes (SOD, CAT) [59,60].

### 2.2. Oncologic Diseases

As demonstrated in various types of human cancers, reactive species are involved in multiple stages of carcinogenesis, including preneoplastic events driven by chronic inflammation, oxidative DNA mutations, proto-oncogene activation, neoplastic cell proliferation, invasion, angiogenesis, and metastasis [3,12,18,63,64,65]. The relationship between OS and cancer is complex, as ROS can both contribute to and result from tumorigenesis. Additionally, ROS can trigger cell death pathways, such as apoptosis and ferroptosis, which may prevent neoplastic events [3,18,66]. In light of these intricate phenomena, a therapeutic approach can be challenging. While enhancing antioxidant defenses might seem appropriate, many chemotherapeutic drugs and radiation therapies actually work by increasing OS in neoplastic cells to induce apoptosis [3,18,67]. However, this strategy also carries the risk of inducing toxic effects in normal tissues [18].

The role of OS in canine oncology has been studied in dogs with various types of cancer, particularly mammary gland tumors [64,65,66,68,69,70,71,72,73], lymphoma [74,75,76,77,78], and mast cell tumors [63,71], among others [71,79,80,81].

OS has been evidenced in dogs with mammary gland tumors, although its manifestation in OS biomarkers appears to depend on the type of sample analyzed. Consistently elevated markers of lipid (i.e., MDA, LOOH) and DNA (i.e., 8-OHdG) oxidation, along with significant alterations in various enzymatic and non-enzymatic antioxidants, have been detected in neoplastic mammary tissue compared to normal mammary gland tissue [64,66,68,69]. Conversely, some studies have reported significant variations in serum or plasma biomarkers in these dogs [70,71], while others have not observed such changes in circulating markers [66,72,73]. Therefore, some researchers recommend direct measurement in target tissues or the collection of multiple blood samples at different time points to account for the detoxification effect on circulating levels [66]. Additionally, a recent study found that female dogs with mammary cancer who received ozone therapy alongside chemotherapy (carboplatin) had a better oxidative profile compared to those receiving standard chemotherapy alone [65].

The literature on OS in canine lymphoma is limited but has produced interesting findings. Studies have reported an altered antioxidant balance in dogs with multicentric lymphoma, as indicated by changes in circulating markers of oxidation (ROS, MDA, and AOPP) and antioxidant defense (antioxidant capacity indexes, vitamins, and enzymes) [71,74,76,77,78]. Notably, two studies observed a correlation between higher OS levels and more aggressive lymphoma characteristics, such as advanced stages (IV and V) and T immunophenotype [74,78]. The impact of treatment on OS remains unclear, potentially due to variations in chemotherapy protocols or the specific biomarkers studied. While some studies have reported a significant correlation between the improvement in OS markers and better clinical response [77,78], others have not found such a correlation [76]. Interestingly, Bottari and colleagues reported even higher circulating markers of oxidation (MDA and AOPP) after CHOP chemotherapy (cyclophosphamide, vincristine, doxorubicin, and prednisone) in dogs with multicentric lymphoma, suggesting that the treatment might exacerbate OS levels [74]. A transient increase in ROS concentrations has also been observed in canine lymphoma and leukemia cell cultures after treatment with benzyl isothiocyanate, suggesting a therapeutic approach targeting OS in these cancers [75].

Lastly, one study revealed increased circulating MDA concentrations in a heterogeneous group of cancer-bearing dogs compared to the control, and another study showed elevated d-ROMs levels and decreased antioxidant capacity in dogs with mast cell tumors [63]. Conversely, another study dismissed the diagnostic value of IsoP for detecting canine urothelial carcinoma [81]. Recent research has also explored the potential of compounds like tepoxalin and myricetin to induce ROS generation and subsequent apoptosis in canine osteosarcoma cell lines [79,80].

### 2.3. Gastrointestinal and Exocrine Pancreatic Diseases

Remarkable evidence suggests that OS plays a significant part in both acute and chronic canine enteropathies, particularly in Inflammatory Bowel Disease (IBD). This is due to the release of reactive species by leukocytes in the inflamed intestinal mucosa and impaired tissue perfusion. Such OS can lead to further cellular damage, the perpetuation of inflammation, and delayed recovery time [82,83,84,85,86,87]. Consequently, OS-derived molecules have been proposed as promising biomarkers for canine enteropathies. Studies have consistently reported elevated levels of various oxidation biomarkers in the serum or plasma of dogs with IBD and acute enteropathies, including ROS, d-ROMs, MDA, and IsoP, with some also correlating with the severity of clinical presentation [82,85]. Notably, dogs with IBD often exhibit lower levels of several antioxidant biomarkers, such as TAS, CUPRAC, FRAP, and thiol groups [85,86,87]. Interestingly, Minamoto et al. [84] employed a comprehensive untargeted metabolomic approach and identified a significant impact of OS in canine IBD, which persisted even in dogs with apparent clinical improvement. A recent study investigating the response to treatment with allogeneic mesenchymal stem cells in these dogs did not observe changes in MDA levels but proposed albumin as an alternative antioxidant marker in IBD [83]; this has also been supported by the recently reported association between TAS and albumin in dogs [23]. These observations highlight the potential therapeutic value of antioxidant supplementation as a supportive or alternative approach to antimicrobial treatment in canine enteropathies, although further clinical trials are warranted [82,83].

OS has also been proposed to participate in the pathogenesis of canine pancreatitis, although research in this area has been more limited [88,89]. A recent study found elevated levels of reactive metabolites in dogs with acute pancreatitis and identified a significant correlation between urinary IsoP, C-reactive protein, and canine-specific pancreatic lipase [89]. These findings suggest a potential link between OS, pathological calcium signaling, mitochondrial dysfunction, and the amplification of inflammation in canine pancreatitis through ROS. However, the exact mechanisms underlying this relationship require further investigation [88,89].

### 2.4. Hepatobiliary Diseases

The liver plays a central role in redox regulation, making it both a major producer of reactive species and a target for their damaging effects. This vulnerability arises from the liver’s crucial functions in metabolism and toxin biotransformation, which contribute significantly to reactive species production [90,91]. Notably, copper metabolism is a significant source of ROS in the liver, and its dysregulation can contribute to the development of hepatitis and cirrhosis [92]. Additionally, the liver is the primary site for synthesizing GSH, considered the major intracellular antioxidant [90,93].

The implications of OS in hepatic diseases in dogs have been studied through the quantification of oxidants and antioxidants in various samples, such as blood, urine, and liver tissue, using a range of methods [90,91,92,93,94,95,96,97,98,99,100,101,102,103,104]. Elevated urinary IsoP levels have been found in dogs with liver disease of various origins, with a particularly pronounced increase in those with congenital portosystemic shunts [94,100]. Increased levels of plasmatic reactive metabolites and higher immunohistochemical expression of MDA in liver tissue have been reported in dogs with chronic hepatitis and copper-associated hepatitis. These markers have also shown a significant correlation with copper accumulation, necroinflammatory activity, and fibrosis scores [102,103]. The literature on impaired antioxidant defense due to the hepatic depletion of GSH dogs is extensive. Low reduced and oxidized glutathione ratios (GSH/GSSG) are often found in dogs with various hepatopathies (i.e., necroinflammatory liver disorders, extrahepatic bile duct obstruction, copper toxicosis, chronic extrahepatic cholestasis, and chronic hepatitis), along with decreased values of antioxidant enzymes and total antioxidant capacity indexes [90,91,92,102,104]. Further support for these findings comes from transcriptome and gene array analyses of liver tissue from dogs with hepatitis and age-related hepatic changes, which suggest the enhanced expression of genes related to OS and inflammation in hepatic dysfunction [95,99]. Overall, OS biomarkers appear to be promising for assessing canine liver disease of various origins, with only a few studies reporting differently [94,97,102].

Consequently, several authors advocate for antioxidant therapeutic interventions in canine hepatopathies, and various supplements have traditionally been included in their medical management [91,93,94,96,98]. However, the efficacy of antioxidant administration in these patients remains a topic of debate [91,94,100]. Given the complexity of redox homeostasis, supplementing with a single antioxidant may not sufficiently alter OS biomarkers to be detectable by statistical analysis [91]. Traditionally, therapeutic approaches have focused on replenishing depleted GSH [93,94] by administering glutathione precursors like S-adenosylmethionine (SAMe), which is more readily available for clinical practice. Other antioxidant products with evidence of efficacy in canine hepatopathies include vitamin E, ursodeoxycholic acid, and extracts of the milk thistle plant (Silymarin, Silybin, and Silybinin), among others [91,93,96,98]. Webb and Twedt’s review provides recommended dosages for these products in dogs and suggests the potential benefits of combination therapy [91].

### 2.5. Endocrine Diseases and Obesity

The contribution of OS to canine endocrinopathies has been studied mainly in dogs with hypothyroidism [105,106,107], Cushing’s syndrome [108,109,110], diabetes [111,112,113,114], obesity [115,116,117,118,119], and hyperlipidemia [120].

Data on hypothyroidism and OS, both in humans and dogs, are particularly conflicting. Thyroid hormones significantly influence redox homeostasis, yet their specific effects in hyper- and hypothyroidism remain intriguing, as discussed in a previous review [121]. Various studies have examined comprehensive panels of biomarkers in hypothyroid dogs, suggesting that increased OS is present, although the results vary widely depending on the biomarker studied and the sample type. Some studies have reported elevated d-ROMs and MDA levels in the blood and saliva of hypothyroid dogs [105,106,107], while others have found lower AOPP levels [105,106]. Similarly, the literature shows both increased [105,107] and decreased [106] levels of several antioxidant indexes. In this context, sample type seems to be relevant, with whole-blood samples potentially providing a better reflection of the altered redox homeostasis in these patients [106].

Unlike the well-established link between increased OS and diabetes in humans, which is associated with dysfunctional mitochondria and glucose auto-oxidation [3,111], studies in dogs have produced variable results. Some studies have reported increased markers of lipid and DNA oxidation, particularly in poorly controlled diabetic dogs, suggesting an association with disease severity [111,113]. Additionally, significant improvements in OS biomarkers have been observed following antioxidant supplementation with N-acetylcysteine [113] and Fibroblast growth factor-21 [112]. Conversely, a recent study found no differences in MDA or SOD levels between diabetic and control dogs and did not observe a significant benefit of another antioxidant, Andrographis paniculata [114].

Regarding Cushing’s syndrome, research in canine species has been limited but consistent. Two studies reported increased markers of lipid and protein oxidation in dogs with hypercortisolism, particularly in poorly controlled patients. These studies also noted a significant reduction in oxidation markers following treatment, highlighting both the presence of increased OS and the significant benefits of medical control in these patients [108,109].

A reasonable question arises when evaluating OS, particularly lipid peroxidation, in dogs with endocrinopathies (e.g., Cushing’s syndrome and hypothyroidism): the potential influence of the patient’s body condition score in the results [107,109]. Since obesity in humans has been associated with chronic inflammation and increased OS, several studies have studied this relationship in dogs [115,116,117,118,119]. Although this aspect remains unclear [118,119], significant variations in OS biomarkers have been detected in the blood, saliva, and adipose tissue of obese dogs [115,116,117,119]. Additionally, the impact of hyperlipidemia [120] and the significant interference of lipemia with OS biomarkers such as MDA and TAS have been reported [122]. This interference should be considered when interpreting results from dogs with endocrine diseases.

### 2.6. Hematologic Diseases

Red blood cells (RBCs) are particularly vulnerable to ROS attacks due to their ubiquity, proximity to oxygen molecules, and elevated iron concentrations. In humans, OS has been proposed as both a cause and consequence of anemia through mechanisms such as reducing the mean lifespan of RBCs or increasing tissue oxygen demand and ROS production [123,124,125]. While the literature on this topic in dogs is scarce, it has provided some interesting data on anemia of various causes: hemolytic, non-hemolytic, secondary to kidney, infectious, and oncologic diseases, among others [124,125,126,127,128]. Studies have shown variable changes in oxidation markers (ROS, IsoP, and MDA) [124,125,126], but substantial evidence suggests that OS and antioxidant depletion are involved in the development of anemia in dogs. This link is likely due to decreased antioxidant defenses, both enzymatic and non-enzymatic (GPX, Vitamin E, and GSH) [125,126,127,128]. Further investigation is needed to determine whether OS is a primary cause or a secondary effect of anemia and explore if antioxidant therapy could improve survival and overall outcomes in dogs with anemia from various causes [125,126,127]. It is also important to consider that hemolysis and icterus can be potential sources of interference, frequently affecting plasma or serum samples from these patients [122].

Additionally, two studies have highlighted that OS could be a significant concern in canine hemotherapy. The accumulation of oxidation products (MDA, PC) and depletion of natural cellular antioxidants (TAS, SOD, GPX, and CAT) have been detected in stored canine whole blood, along with increased hemolysis. These findings suggest that prolonged storage periods (>28 days) might discourage the use of stored blood in certain cases [129]. Furthermore, the therapeutic efficacy of canine bone marrow mesenchymal stem cell transplantation may be compromised by OS-mediated senescence, as indicated by increased levels of ROS and decreased antioxidant enzymes. This effect has been mitigated by adding antioxidants, such as mitoquinone, to cell cultures [130].

### 2.7. Infectious and Parasitic Diseases

#### 2.7.1. Vector-Borne Diseases

##### Leishmaniosis

The role of OS in the pathogenesis of canine leishmaniosis (CanL) has been extensively studied, and substantial evidence has been gathered on the relevance of OS and its association with the clinical stages of CanL [131,132,133,134,135,136,137,138,139,140,141,142,143]. The interplay between the host’s immune response, the parasites, and OS creates a complex pathogenic landscape.

While *Leishmania* spp. can initially evade the immune system by suppressing ROS production by phagocytes, the subsequent development of inflammation in CanL is characterized by an increased influx of activated neutrophils and macrophages that generate high levels of oxidants. This contributes to the progression of the disease and a concomitant weakening of antioxidant defenses [131,133,134,139,140]. These aspects are supported by several studies that have found increased circulating oxidation markers (e.g., ROS, MDA, and total oxidant status) [132,133,134,135,137,138] and variable changes in antioxidant markers (e.g., TAS, CUPRAC, FRAP, GSH, and thiol groups) depending on the clinical stage [133,134,137,140,141,142]. Almeida and colleagues also found that OS in CanL causes neutrophil dysfunction, leading to their apoptosis, particularly in severe stages and in association with uremia [132,133]. More recent findings have suggested that increased OS impairs the lymphoproliferative response and, therefore, cellular immunity in dogs with CanL [134]. Additionally, correlations between OS biomarkers and parasite load have been observed [142], as well as improvements in antioxidant defense following successful therapy, indicating that OS may be a useful tool for monitoring the treatment and clinical follow-up of sick dogs [140].

Despite the established link between OS and disease progression in CanL, therapeutic strategies targeting the redox state have not been extensively explored. While some authors advocate for tailoring CanL treatment plans based on the patient’s redox status [139], research on the efficacy of enhancing the antioxidant defense system in this disease remains limited. A recent study reported a decrease in circulating MDA and PC, along with an increase in GSH, after the addition of nutritional adjuvants (omega-3 PUFAs and B vitamins) to standard anti-Leishmania treatment [136]. However, further research on this topic is needed.

##### Ehrlichiosis

Significant alterations in redox status have been documented in canine ehrlichiosis [144,145,146,147,148,149,150,151,152]. Increased levels of ROS, MDA, and AOPP have been observed in both naturally and experimentally infected dogs [144,145,146,147,149,151], while a decrease in nitric oxide (NO) and MDA was noted following doxycycline treatment [149]. Antioxidant markers (e.g., TAS, CUPRAC, FRAP, GPX, thiol groups, and others) have shown either increases or decreases depending on the disease stage (acute versus subclinical). These fluctuations likely reflect the complex interplay between OS and infectious agents [144,146,147,150,151].

##### Babesiosis

Studies on OS canine babesiosis have consistently found elevated levels of reactive species (NO), lipid (MDA), and DNA oxidation markers (8-OHdG), along with variable alterations in antioxidant enzymes and indexes (e.g., TAS, SOD, CAT, and GPX) in infected dogs [145,148,153,154,155,156,157,158]. Various authors have proposed that OS could be one of the mechanisms leading to anemia in dogs with babesiosis, as a result of oxidative damage to erythrocytes, favoring their destruction [155,156,158]. Additionally, infected dogs with secondary multiple organ dysfunction have shown more pronounced redox alterations, suggesting OS biomarkers could serve as indicators of disease severity and outcomes in canine babesiosis [155].

##### Other Vector-Borne Diseases

Similar trends of increased DNA and lipid oxidation have been observed in dogs with heartworm disease, alongside variable findings in antioxidant markers [159,160,161]. Additionally, OS has been proposed to play a role in the pathogenesis of canine hepatozoonosis and trypanosomosis and may be related to the development of anemia due to increased lipid peroxidation in erythrocytes [162,163].

#### 2.7.2. Infectious and Parasitic Gastrointestinal Diseases

Canine parvoviral enteritis is associated with OS, as evidenced by increased circulating MDA and NO levels, along with alterations in enzymatic and non-enzymatic antioxidant markers, likely due to the virus-induced release of pro-inflammatory cytokines [164,165,166,167]. The addition of antioxidants such as N-acetylcysteine, resveratrol, and vitamin C to standard therapy can reduce the concentrations of MDA and NO and enhance the activity of certain antioxidant enzymes. However, a clear improvement in clinical scores or survival rates following antioxidant therapy has not been consistently demonstrated, and further research is necessary to determine the optimal selection and dosage of antioxidants for this purpose [164,165].

Regarding gastrointestinal parasites, the role of OS in their pathogenesis remains unclear. A study found significant changes in ROS metabolites and thiol levels in dogs with gastrointestinal nematodosis [168], while another study failed to demonstrate alterations in antioxidant markers in parasitized dogs [169].

#### 2.7.3. Ectoparasites and Dermal Fungal Diseases

Canine demodicosis [170,171,172,173,174] and sarcoptic mange [175,176,177,178] are associated with increased OS. This is believed to result from the presence of parasites in the skin, which release antigenic material and trigger the production of pro-inflammatory cytokines. These factors may contribute to pathological changes in the tissue, such as erythema, edema, hypersensitivity, pruritus, and hyperkeratosis [173,174,175,176]. Elevated levels of peripheral oxidation biomarkers (MDA and other lipid hydroperoxides) have been consistently observed in dogs with both localized and generalized demodicosis, as well as in those with sarcoptic mange [170,171,173,175,176,177,178]. Markers of antioxidant defense have shown variable changes: while most of the studies have reported significant depletions in antioxidants like SOD, CAT, GPX, and vitamins [170,175,176,178], others have found no change or even increased levels in infested dogs compared to control [171,172,173,177]. This variability might be explained by an initial upregulation of antioxidant defenses, followed by their overutilization or sequestration in the skin as the disease progresses [170]. Interestingly, some authors have identified a relationship between OS, the severity of the infestation, and the rate of apoptosis in peripheral leukocytes in dogs with sarcoptic mange [176,178]. Moreover, treatment with ivermectin appears to normalize OS markers in both demodicosis and sarcoptic mange, especially when antioxidants such as vitamin E and selenium are added to standard therapy [172,175]. Additionally, a study reported increased OS in canine dermatophytosis, specifically noting a rise in circulating MDA and a decrease in both enzymatic and non-enzymatic antioxidants [179].

### 2.8. Neurologic Diseases

The nervous system, particularly the brain, is highly vulnerable to oxidative damage due to its high energy and oxygen consumption, the large concentration of PUFAs in myelin membranes, and its relatively low antioxidant defenses [180,181]. While OS has been linked to the etiopathology of several neurologic diseases in humans [181], research in dogs remains limited. A recent study in dogs with idiopathic epilepsy, experiencing either focal or generalized seizures, revealed significant alterations in circulating OS biomarkers, including higher levels of AOPP and lower levels of GSH, thiol groups, and other antioxidants [182]. These findings may be attributed to neuroinflammation and accelerated ROS-mediated neuronal deterioration, which could induce subsequent seizures [182,183]. Furthermore, Marquis et al. [180] evaluated IsoP, acrolein, and GSH levels in urine, cerebrospinal fluid, and spinal cord tissue of dogs with ascending–descending myelomalacia following spinal cord injury, finding exacerbated OS and a potential association with neurodegeneration and necrosis. In contrast, while the role of OS in canine motor neuron disease and degenerative myelopathy in Pembroke Welsh Corgi dogs has been studied, it has not been fully clarified [184,185,186].

### 2.9. Renal Diseases

Renal cells, particularly tubular epithelial cells, are significant sources of endogenous ROS due to their high mitochondrial activity, arterial blood flow, and the activity of ROS-producing NOX family enzymes [187,188]. Increases in renal ROS production can lead to the release of pro-inflammatory cytokines, and, if persistent, to inflammation and renal fibrosis, making OS a proven contributing factor to Chronic Kidney Disease (CKD) in both human and animal models [187,188,189,190]. This issue becomes even more concerning when the few remaining nephrons become hyperfunctional, further increasing mitochondrial oxidative phosphorylation and ROS production [187,189,190]. Additionally, several factors commonly present in humans and animals with CKD can exacerbate OS, including the activation of the renin–angiotensin system, systemic hypertension, chronic inflammation, proteinuria, anemia, and advanced age [187,190].

Various authors have studied the implications of OS in dogs with renal disease [124,187,189,190,191,192,193,194,195,196]. With very few exceptions in some circulating antioxidant indexes [189], most studies on canine CKD have evidenced significant alterations in OS biomarkers, especially in MDA, IsoP, ROS, TAS, and antioxidant enzymes [124,191,195,196]. Some studies have also found significant correlations between MDA, creatinine concentration [124], and the degree of renal dysfunction [192]. The role of OS has also been observed in nephrotoxicity caused by hemoglobinuria [193], chemotherapeutic drugs (cisplatin) [194], and uremic toxins (methylguanidine) in dogs [191]. Additionally, it has been shown that OS accelerates neutrophil apoptosis in canine CKD, potentially affecting their innate immune response [191,195]. Protecting the kidney from OS through antioxidant supplementation and other therapeutic actions has been suggested in dogs, as summarized in Brown’s review [187]. However, further clinical investigations are warranted due to the limited research in this area [187,190].

### 2.10. Dermatologic Diseases

The skin is continuously exposed to reactive species from both endogenous and environmental sources, necessitating robust enzymatic and non-enzymatic antioxidants, such as vitamins and carotenes [197,198]. Similar to humans, altered dermal redox homeostasis in dogs has been linked to certain skin diseases, particularly atopic dermatitis [198,199,200,201,202]. Despite some discrepancies existing depending on the specific biomarkers used, several studies have demonstrated a correlation between clinical scores (i.e., Canine Atopic Dermatitis Extent and Severity Index, CADESI) and OS biomarkers like MDA, antioxidant enzymes, and vitamin E [198,199,200]. The contribution of OS to atopic dermatitis is likely related to the infiltration of the skin with inflammatory cells and cytokines, which promote ROS formation and disrupt the skin’s antioxidant barrier [197,199,200]. Consequently, OS biomarkers have been proposed as useful tools for precision medicine in dogs with atopic dermatitis [199]. Furthermore, various researchers have advocated for a multimodal therapeutic approach that includes nutritional interventions and antioxidant supplementation (e.g., vitamins and carotenes) alongside standard therapies [198,199,202]. Limited data suggest that OS might also play a role in canine zinc-responsive dermatosis, although further investigation is warranted [203].

### 2.11. Ophthalmologic Diseases

OS is considered a risk factor for eye diseases [204] and has been studied primarily in two ophthalmologic disorders in dogs: cataracts [205,206,207,208,209,210,211] and glaucoma [212,213,214,215].

Lenses are chronically exposed to photo-oxidation of their proteins and lipids due to UV radiation, leading to protein aggregation and, ultimately, lens opacification. Despite the presence of antioxidant agents within the lens (such as vitamins and antioxidant enzymes), OS is widely recognized as a major contributor to cataract development, alongside other environmental and endogenous factors [205,206,210,211]. Significant alterations in oxidative biomarkers (MDA) and antioxidant biomarkers (TAS, SOD, CAT, and GPX) have been detected in the blood and aqueous humor of cataractous dogs [207,209]. Additionally, decreased antioxidant capacity and vitamin C levels have been observed in the aqueous humor of dogs following extracapsular lens extraction and experimental phacoemulsification, suggesting that these surgical procedures initially induce an OS condition in the eye [206,208]. In attempting to prevent or delay cataract formation, both oral antioxidant supplements and topical antioxidant eye drops have been used in humans and dogs [210,211]. Examples of antioxidant agents demonstrating protective effects in dogs, particularly in incipient cataracts, include grape seed extracts, vitamins C and E, curcuminoids, and others [205,210].

Similarly, OS appears to be a major contributor to retinal ganglion cell degeneration and glaucoma development [212,214,215]. Increased immunolabeling for OS biomarkers has been observed in the retinal tissue of dogs with acute glaucoma [213], and lower antioxidant enzymes (GPX) have been related to an increased risk of inherited glaucoma in Euraiser dogs [212]. Although antioxidants have been proposed to protect canine retinal membranes under experimental conditions [204], the literature on this topic remains limited.

### 2.12. Orthopedic Diseases

Reactive species are considered important mediators in the pathophysiology of osteoarthritis. Chondrocytes and activated inflammatory cells in this condition release increased amounts of ROS, which further damage collagen, proteoglycans, and hyaluronic acid and enhance chondrocyte senescence and cartilage degradation [216,217,218,219,220]. This redox imbalance has been documented in dogs with both naturally occurring and experimentally induced osteoarthritis, as evidenced by OS biomarkers in blood and canine chondrocyte cell cultures [217,218,219,220,221]. Enhanced oxidative processes have also been observed in circulating OS biomarkers in dogs suffering from hip dysplasia, likely due to similar mechanisms of cartilage inflammation and degradation [216,222].

Dietary composition, particularly the lipid profile with a focus on omega-3 PUFAs and eicosapentaenoic acid (EPA), appears to play a critical role in mitigating these processes. Both pharmaceutical interventions (e.g., N-acetylcysteine) [218] and nutraceutical products (e.g., fish oil, corn oil, and other plant-derived compounds) [217,220,221] have demonstrated protective effects against OS in canine osteoarthritis.

### 2.13. Reproductive System Diseases

Recent studies have shown consistent alterations in OS biomarkers measured in blood, urine, and uterine tissue in bitches with cystic endometrial hyperplasia and pyometra. These findings suggest that excessive ROS production may be a significant factor contributing to uterine damage by weakening local antioxidant defenses and exacerbating these disorders [223,224,225]. Additionally, as summarized in Domosławska-Wyderska and colleagues’ recent review [226], various studies indicate that OS may play a relevant role in the pathogenesis of canine benign prostatic hyperplasia. This association could be linked to age-related hormonal changes and chronic inflammation of the prostate. However, further research is needed to evaluate the potential benefits of antioxidants in this condition [226,227].

### 2.14. Dental Diseases

Studies investigating OS markers in canine periodontal disease have yielded mixed results [228,229]. While one recent study found no changes in salivary MDA concentrations [229], a previous study detected a significant accumulation of MDA and 8-OHdG in the saliva of dogs with periodontal disease, along with an increase in salivary SOD activity [228]. This earlier study also found correlations between OS biomarkers and the severity of gum and teeth clinical signs, which were attributed to the inflammatory processes in the oral cavity [228].

### 2.15. Others

Additionally, other studies have demonstrated that OS is present in dogs with ischemia-reperfusion injury [230], as well as in systemically ill dogs undergoing hospitalization due to various underlying disorders (e.g., infectious, inflammatory, immune-mediated, metabolic, and neoplastic) [231,232]. It has been observed that hospitalized dogs exhibit increased lipid peroxidation (elevated urinary IsoP levels) and antioxidant depletion, particularly in GSH and vitamin E. While N-acetylcysteine supplementation did not appear to improve overall redox state in these dogs, further research is needed to explore other antioxidant therapeutic options and their impact on longer-term outcomes [231,232].

## 3. Conclusions

Solid evidence demonstrates the role of OS in a multitude of canine diseases, impacting diverse organs and systems. In some conditions, it remains unclear whether reactive species are significant causative agents or merely byproducts of the inflammatory processes involved. Inconsistencies across studies may arise from differences in sample selection, the specific OS biomarkers used, and variations in analytical methods. Moreover, interpreting increased antioxidant defenses as a response to OS or antioxidant depletion as a sign of imbalance can be challenging.

Therapeutic approaches to managing OS vary widely among canine diseases. Certain antioxidants are commonly used in some diseases (e.g., hepatopathies), while pro-oxidant drugs are employed in others (e.g., oncology). In some areas, this issue remains underexplored.

To our knowledge, this is the first comprehensive review summarizing the current understanding of OS in canine pathology, with the aim of paving the way for further research in such a broad and evolving field.

**Table 2:** Selected studies on oxidative stress in canine diseases.

Group	Sub-Group	Disease *	Biomarkers of Oxidation*	Biomarkers of Antioxidant Defense *	Sample Type	Reference
Cardiovascular, respiratory, and related diseases	Cardiovascular	MMVD	MDA, mtDNA	-	Blood	[32]
		MMVD	MDA, OxLDL	Vitamin E	Blood	[39]
		MMVD and DCM	MDA	GPX, Vitamin E	Blood	[42]
		MMVD and DCM	MDA	GPX	Blood	[38]
		MMVD	-	CUPRAC, SOD, CAT, GR	Blood	[36]
		MMVD and DCM	-	TAS (ABTS), CUPRAC, Thiol	Blood	[40]
		MMVD, DCM, and others (Heart Failure)	-	TAS (ABTS), SOD, CAT, GPX	Blood	[37]
		MMVD stage B1	MDA	SOD, GPX, Vitamin E	Blood	[41]
		MMVD and DCM (Heart Failure)	MDA, IsoP, PC	GSH:GSSG, vitamins A, C, and E, ORAC	Blood	[35]
		DCM	MDA	GPX, SOD, Vitamins A, C, E	Blood	[34]
		DCM	-	GPX, SOD, Vitamins A, C, E	Blood	[33]
		MMVD and Heart Failure	-	-	Review	[43]
		MMVD	MDA	-	Blood	[44]
		MMVD	IsoP	GPX	Blood	[46]
		MMVD	IsoP	-	Blood	[45]
	Experimental cardiac models	Induced atrial fibrillation	ROS, XO	GPX, SOD	Blood	[52]
		Induced atrial fibrillation	ROS	-	Cardiac tissue	[49]
		Induced atrial fibrillation	ROS, XO	-	Blood	[51]
		Induced atrial fibrillation	ROS	-	Cardiac tissue	[47]
		Induced atrial fibrillation	ROS	-	Cardiac tissue	[50]
		Induced atrial fibrillation	ROS, 8-OHdG	-	Cardiac tissue	[48]
		Induced heart failure	Panel of aldehydes	-	Cardiac tissue	[55]
		Induced cardiac arrest	IsoP	Panel of enzymes	Cardiac tissue	[54]
		Induced heart failure	Panel of aldehydes	-	Cardiac tissue	[56]
		Induced cardiac arrest	IsoP	-	Coronary sinus plasma	[53]
	Respiratory	Tracheal collapse	MDA	-	Blood	[57]
		Tracheal collapse	MDA	-	Blood	[61]
		Air pollution	MDA, NO	SOD, CAT, GSH, SOD	Blood	[59]
		Brachycephalic Obstructive Airway Syndrome	MDA	SOD, GPX	Blood	[58]
		Chromium pollution	MDA	SOD, CAT	Tissues	[60]
		Hypoxia-induced neurogenic pulmonary edema	MDA, PC	-	Tissues	[62]
Oncologic diseases	Mammary gland tumors	Mammary gland tumors	MDA	-	Blood	[72]
		Mammary gland tumors	MDA	TAS (ABTS)	Blood	[65]
		Mammary gland tumors	MDA, 8-OHdG	-	Mammary gland tissue	[68]
		Mammary gland tumors	MDA	GSH, G6PD	Mammary gland tissue	[64]
		Mammary gland tumors	NO, AOPP	FRAP	Blood	[70]
		Mammary gland tumors	MDA	Vitamin E	Blood and mammary gland tissue	[66]
		Mammary gland tumors	MDA, LOOH	SOD, CAT, GSH, GST, Vitamin C	Mammary gland tissue	[69]
		Mammary gland tumors	MDA	SOD, GPX, Thiol	Blood	[73]
	Lymphoma and leukemia	Lymphoma and lymphoid leukemia	ROS	-	Cell culture	[75]
		Lymphoma	d-ROMs	BAP	Blood	[76]
		Lymphoma	MDA, AOPP	FRAP	Blood	[74]
		Lymphoma	MDA, IsoP	ORAC, GPX, Vitamin C, Vitamin E	Blood	[78]
		Lymphoma	MDA, ROS	GSH:GSSG, GPX, FRAP, SOD	Blood and lymph node tissue	[77]
	Other oncologic diseases	Osteosarcoma	ROS	-	Neoplastic cells	[80]
		Osteosarcoma	ROS	-	Neoplastic cells	[79]
		Mast cell tumor	d-ROMs	BAP, Vitamin E	Blood	[63]
		Urothelial carcinoma	IsoP	-	Urine	[81]
		Various cancer types: Mammary gland carcinoma, mast cell tumor, osteosarcoma, and others.	MDA	-	Blood	[71]
Gastrointestinal and exocrine pancreatic diseases	Gastrointestinal diseases	Chronic inflammatory enteropathy	MDA	GSH, Albumin	Blood	[83]
		Acute Diarrhea (non-specific acute enteropathies)	d-ROMs, OSI	SAC	Blood	[82]
		IBD	ROS, MDA, FOX	TAS, CUPRAC, FRAP, Thiol, PON-1	Blood	[85]
		IBD	-	CUPRAC	Blood	[87]
		IBD	-	TAS (ABTS)	Blood	[86]
		IBD	Metabolomic profile	Metabolomic profile	Blood	[84]
	Exocrine pancreatic diseases	Acute Pancreatitis	RM, IsoP	AOP	Blood, urine	[89]
		Pancreatitis	-	-	Review	[88]
Hepatobiliary diseases		Acute liver injury	MDA, H_2_O_2_, 8-OHdG	G6PD, TrxR, CAT, SOD, GPX, GR, GSH	Liver tissue	[98]
		Liver disease (various origins)	-	GSH	Blood	[93]
		Liver disease (various origins)	IsoP	-	Urine	[100]
		Liver disease	d-ROMs	Thiol	Blood	[96]
		Liver injury	ROS	CAT, GPX	Liver tissue	[104]
		Chronic hepatitis	MDA, 4-HNE	-	Liver tissue	[103]
		Cooper-associated hepatitis	RM, IsoP	TAS (ABTS)	Blood and urine	[102]
		Liver disease (various origins)	IsoP	GSH, Vitamin E	Blood, urine, and liver tissue.	[94]
		Cooper-associated hepatitis	Transcriptome and gene Arrays	Transcriptome and gene Arrays	Liver tissue	[95]
		Chronic liver disease	MDA	-	Liver tissue	[101]
		Age-related hepatic alterations	Genome Arrays	Genome Arrays	Liver tissue	[99]
		Liver disease	-	-	Review	[91]
		Portosystemic Shunt	-	Vitamin C	Blood	[97]
		Liver disease (various origins)	-	GSH/GSSG and antioxidants gene expression	Liver tissue	[92]
		Liver disease (various origins)	-	GSH/GSSG	Liver tissue	[90]
Endocrine diseases and obesity	Hypothyroidism	Hypothyroidism	TOS, POX-Act, d-ROMs, AOPP, MDA	CUPRAC, FRAP, TAS (ABTS), PON-1	Blood	[106]
		Hypothyroidism	MDA	TAC	Blood	[107]
		Hypothyroidism	MDA, d-ROMs, TOS, POX-Act, AOPP	CUPRAC, FRAP, TAS (ABTS), Thiol, PON-1, GPX, FRAS	Blood and saliva	[105]
	Cushing’s syndrome	Cardiac fibrosis-Cushing’s syndrome	8-OHdG, NADPH oxidase	SOD	Blood and cardiac tissue	[110]
		Cushing’s syndrome	MDA	-	Blood	[109]
		Cushing’s syndrome	PC	-	Blood	[108]
	Diabetes	Diabetes	MDA	SOD	Blood	[114]
		Diabetes	H_2_O_2_, 8-OHdG, MDA	CAT, SOD, GPX, GSH-GSSG, TrxR, NADPH-NADP+, Thiol	Cerebrum tissue	[113]
		Diabetes	ROS, MDA	CAT, GPX, GR, SOD	Pancreatic tissue	[112]
		Diabetes	MDA	CAT, GSH	Blood	[111]
	Hyperlipidemia	Hyperlipidemia	MDA	-	Blood	[120]
	Obesity	Obesity	MDA	-	Blood	[115]
		Obesity	MDA, ROS, FOX	CUPRAC, FRAP, TAS (ABTS), Thiol, PON-1	Blood	[119]
		Obesity-related metabolic dysfunction	-	Proteomics	Saliva	[117]
		Obesity	MDA	FRAP, Ceruloplasmin	Blood	[118]
		Obesity	-	Transcriptomics	Blood, adipose tissue	[116]
Hematologic diseases	Hemotherapy	Stored blood (transfusion medicine)	MDA, PC	TAS (ABTS), SOD, GPX, CAT	Blood	[129]
		Bone marrow mesenchymal stem cells (BMSCs) transplantation	ROS, MDA	SOD, CAT, GPX	BMSCs culture	[130]
	Anemia (various origins)	Anemia (hemolytic and nonhemolytic)	ROS	GSH, Vitamin E	Blood	[125]
		Anemia (various origins)	IsoP	TAS (ABTS), GPX	Blood and urine	[126]
		Anemia (CKD)	MDA	GSH-GSSH, GPX, GR, SOD	Blood	[124]
		Immune-mediated hemolytic anemia	-	Peroxiredoxin-2	Blood	[128]
		Immune-mediated hemolytic anemia	MDA	Vitamin E	Blood	[127]
Infectious and parasitic diseases	Vector-borne diseases	Leishmaniosis	MDA, PC	GSH/GSSG	Blood	[136]
		Leishmaniosis	ROS, RNS, Hydroperoxides	SOD, FRAP	Blood	[139]
		Leishmaniosis	-	PON-1	Blood	[143]
		Leishmaniosis	MDA	GSH/GSSG, Thiol	Blood	[138]
		Leishmaniosis	TOC, MDA	TAS (ABTS)	Blood	[134]
		Leishmaniosis	TOC, MDA	TAC	Blood and tissues	[142]
		Leishmaniosis	-	SOD	Blood	[141]
		Leishmaniosis	TOS	TAS (ABTS), FRAP, CUPRAC, PON-1, Thiol	Blood	[140]
		Leishmaniosis	-	-	Review	[131]
		Leishmaniosis	ROS	-	Blood	[132]
		Leishmaniosis	TOC, MDA	TAS (ABTS), GSH	Blood	[133]
		Leishmaniosis	MDA	TAS (ABTS)	Blood	[137]
		Leishmaniosis	MDA	GSH, Vitamin C	Blood	[135]
		Ehrlichiosis	R-OOHs	OXY, Thiol	Blood	[150]
		Ehrlichiosis	MDA, NO	-	Blood	[149]
		Ehrlichiosis	MDA, NO	TAC, SOD, GPX	Blood	[146]
		Ehrlichiosis	ROS, MDA, FOX	TAS (ABTS), CUPRAC, FRAP	Blood	[151]
		Ehrlichiosis	AOPP	FRAP	Blood	[144]
		Ehrlichiosis	-	TAS (ABTS), PON-1	Blood	[152]
		Ehrlichiosis	MDA, NO, AOPP	GR	Blood	[147]
		Ehrlichiosis and Babesiosis	MDA, NO	-	Blood	[145]
		Ehrlichiosis and Babesiosis	MDA	-	Blood	[148]
		Babesiosis	-	GSH, SOD, CAT	Blood	[158]
		Babesiosis	LPO	SOD, CAT, TAS (ABTS)	Blood	[156]
		Babesiosis	MDA	TAS (ABTS), SOD, CAT, GPX	Blood	[155]
		Babesiosis	8-OHdG, NO	TAS (ABTS)	Blood	[153]
		Babesiosis	MDA	-	Blood	[154]
		Babesiosis	MDA	-	Blood	[157]
		Heartworm disease	Comet assay (DNA oxidation)	-	Blood	[160]
		Heartworm disease	-	TAS (ABTS), GPX, PON-1	Blood	[159]
		Heartworm disease	MDA	SOD, CAT	Blood	[161]
		Hepatozoonosis	MDA, NO	GSH	Blood	[162]
		Trypanosomosis	LPO	TAS (ABTS), SOD, GSH	Blood	[163]
	Infectious and parasitic gastrointestinal diseases	Parvoviral enteritis	MDA, NO	GST	Blood	[164]
		Parvoviral enteritis	MDA, NO	GST	Blood	[165]
		Parvoviral enteritis	-	TAS (ABTS), PON-1	Blood	[166]
		Parvoviral enteritis	MDA	SOD, CAT	Blood	[167]
		Gastrointestinal helminthiasis	R-OOHs	OXY, Thiol	Blood	[168]
		Gastrointestinal helminthiasis	-	TAS (ABTS), PON-1	Blood	[169]
	Ectoparasites and dermal fungal diseases	Demodicosis	MDA	SOD, GPX, TAC, CAT	Blood	[173]
		Sarcoptic mange	MDA	SOD, CAT, vitamin A, vitamin C	Blood	[176]
		Demodicosis	-	-	Review	[174]
		Demodicosis	-	PON-1, TAS (ABTS)	Blood	[172]
		Demodicosis	MDA	SOD, CAT, β-carotene, vitamin C	Blood	[170]
		Sarcoptic mange	MDA	SOC, CAT, GPX, GSH, GST	Blood	[178]
		Sarcoptic mange	MDA	GSH, SOD, CAT	Blood	[175]
		Sarcoptic mange	TOS, LOOH	TAS (ABTS), Thiol	Blood	[177]
		Demodicosis	MDA	GSH, SOD, CAT	Blood	[171]
		Dermatophytosis	MDA	SOD, CAT, β-carotene, vitamin C	Blood	[179]
Neurologic diseases		Epilepsy	-	-	Review	[183]
		Epilepsy	MDA, AOPP	GSH, PON-1, Thiol	Blood	[182]
		Myelomalacia	IsoP, Acrolein	GSH	Urine, cerebrospinal fluid, and spinal cord tissue samples	[180]
		Degenerative Myelopathy, Pembroke Welsh Corgi	NO	SOD	Spinal cord tissue samples	[186]
		Degenerative Myelopathy, Pembroke Welsh Corgi	IsoP	-	Cerebrospinal fluid	[184]
		Hereditary canine spinal muscular atrophy	-	SOD, GPX, Vitamin E	Blood	[185]
Renal diseases		Chronic Kidney Disease	IsoP	-	Urine	[196]
		Nephrotoxicity	MDA, ROS	SOD, CAT	Madin–Darby canine kidney cell culture	[194]
		Chronic Kidney Disease	d-ROMS	-	Blood	[190]
		Chronic Kidney Disease	-	CUPRAC	Blood	[189]
		Chronic Kidney Disease and Nephrotoxicity	MDA, ROS	TAS (ABTS)	Plasma and canine neutrophils	[191]
		Nephrotoxicity	4-HNE, Hb-oxidation products	-	Renal tissue	[193]
		Chronic Kidney Disease	MDA	GSH-GSSH, GPX, GR, SOD	Blood	[124]
		Chronic Kidney Disease	MDA, ROS	TAS (ABTS)	Plasma and canine neutrophils	[195]
		Renal azotemia	MDA	CAT, GSH	Blood and urine	[192]
		Chronic Kidney Disease	-	-	Review	[187]
Dermatologic diseases		Atopic dermatitis	-	-	Clinical Scores	[202]
		Atopic dermatitis	FOX	TAS, CUPRAC, FRAP, Thiol	Blood	[199]
		Atopic dermatitis	MDA	TAC, GPX, SOD, Vitamin E	Blood and skin tissue	[198]
		Atopic dermatitis	-	Vitamin E	Blood and skin tissue	[201]
		Atopic dermatitis	MDA	TAS, GPX, SOD	Blood	[200]
		Zinc-responsive dermatosis	-	SOD, metallothionein, heat shock proteins	Skin tissue	[203]
Ophthalmologic diseases		Cataracts	-	-	Ophthalmologic clinical evaluation	[210]
		Cataracts	MDA	TAS (ABTS)	Blood	[209]
		Cataracts	Western immunoblotting	-	Canine lens epithelial cells	[205]
		Cataracts	-	-	Review	[211]
		Cataracts	-	TAC, Vitamin C	Aqueous humor	[208]
		Cataracts	-	SOD, CAT, GPX, G6PD, Vitamin C	Blood and aqueous humor	[207]
		Cataracts		TAC, Vitamin C	Aqueous humor	[206]
		Glaucoma	-	-	Review	[214]
		Glaucoma	-	-	Review	[215]
		Glaucoma	MDA, Nitrotyrosine	-	Retinal tissue	[213]
		Glaucoma	-	GPX	Blood	[212]
		Retinal oxidative damage	MDA	Vitamin E	Retinal tissue	[204]
Orthopaedic diseases		Osteoarthritis	-	GSH	Blood	[221]
		Osteoarthritis	d-ROMs	OXY, BAP	Blood	[220]
		Osteoarthritis	MDA, 8-OHdG	GSH	Blood	[217]
		Osteoarthritis	-	SOD, GSH	Canine chondrocyte cell culture	[218]
		Osteoarthritis	MDA	CAT	Blood	[219]
		Hip dysplasia	MDA	GSH, CAT, SOD, GPX	Blood	[222]
		Hip dysplasia	MDA	GSH, GPX, SOD, Vitamin E	Blood	[216]
Reproductive system diseases		Cystic endometrial hyperplasia-Pyometra	MDA	SOD, CAT, GPX, GSH, FRAP, TAS (ABTS)	Blood, urine, and uterine tissue	[223]
		Cystic endometrial hyperplasia	TOS, OSI	TAS (ABTS)	Blood	[224]
		Pyometra	-	GSH, Vitamin C	Uterine tissue	[225]
		Benign prostatic hyperplasia	-	-	Review	[226]
		Benign prostatic hyperplasia	Bityrosine, formylkynurenine	FRAP	Blood	[227]
Dental diseases		Periodontal Disease	MDA	-	Saliva	[229]
		Periodontal Disease	MDA, 8-OHdG	FRAP, SOD	Saliva	[228]
Others		Ischemia-reperfusion	-	-	Review	[230]
		Systemically ill hospitalized dogs (various causes)	IsoP	GSH, cysteine, vitamin E	Blood, urine	[231]
		Systemically ill hospitalized dogs (various causes)	IsoP	GSH, cysteine, vitamin E	Blood, urine	[232]

* ABTS: 2,2′-azinobis(3-ethylbenzthiazolin-6-sulfonic acid) test; AOP: antioxidant potential; AOPP: Advanced Oxidation Protein Products; BAP: Biological Antioxidant Potential; BMSCs: Bone marrow mesenchymal stem cells; CAT: Catalase; CKD: Chronic Kidney Disease; CUPRAC: Cupric-Reducing Antioxidant Power; DCM: Dilated Cardiomyopathy; d-ROMs: Reactive Oxygen Metabolites; FOX: Ferrous oxidation-xylenol orange; FRAP: Ferric-Reducing Antioxidant Power; FRAS: ferric-reducing ability of saliva; G6PD: Glucose-6-phosphate dehydrogenase; GPX: Glutathione peroxidase; GR: Glutathione reductase; GSH: Reduced glutathione; GSH:GSSG: Reduced–oxidized glutathione ratio; GST: Glutathione S-transferase; H_2_O_2_: Hydrogen peroxide; IBD: Inflammatory Bowel Disease; IsoP: Isoprostanes; LOOH: Lipid hydroperoxides; MDA: Malondialdehyde; MMVD: Myxomatous mitral valve disease; NO: Nitric oxide; ORAC: Oxygen Radical Antioxidant Capacity; OSI: Oxidative Stress Index; OxLDL: Oxidized low-density lipoprotein; OXY: antioxidant barrier; PC: Protein Carbonyls; PON-1: Paraoxonase 1; POX-Act: Peroxideactivity; RM: Reactive metabolites; R-OOHs: Reactive oxidative metabolites; ROS: Reactive Oxygen Species; SAC: Serum antioxidant capacity; SOD: Superoxide dismutase; TAC: Total Antioxidant Capacity; TAS: Total Antioxidant Status; TOC: Total Oxidant Capacity; TOS: Total Oxidant Status; TrxR: Thioredoxin reductase; XO: Xantine Oxidative; 4-HNE: 4-hydroxy-2-nonenal; 8-OHdG: 8-hydroxy-2′-deoxyguanosine.

## Figures and Tables

**Table 1:** Oxidative stress biomarkers—classification and some examples.

Class	Sub-Class	Examples *
Reactive species	ROS	O_2_^•−^, OH^•^, H_2_O_2_, RO^•^, ROO^•^, O_3_, d-ROMs
	RNS	NO^•^, NO_2_^•^, ONOO^−^
Oxidation biomarkers	Lipids	MDA, 4-HNE, IsoP, ACR
	Nucleic acids	8-OHdG, 8-oxo-Gua
	Proteins	AOPP, PC
Antioxidant biomarkers	Non-enzymatic	Vitamins (A, C, E), metals (Se, Zn), GSH, GSH:GSSG; thiol groups, uric acid
	Enzymatic	GPX, SOD, CAT, GR
	Antioxidant capacity indexes	TAS (ABTS), TAC, CUPRAC, FRAP, TRAP, ORAC, BAP

* ABTS: 2,2′-azinobis(3-ethylbenzthiazolin-6-sulfonic acid) test; ACR: Acrolein; AOPP: Advanced Oxidation Protein Products; BAP: Biological Antioxidant Potential; CAT: Catalase; CUPRAC: Cupric-Reducing Antioxidant Power; FRAP: Ferric-Reducing Antioxidant Power; GPX: Glutathione peroxidase; GR: Glutathione reductase; GSH: Reduced glutathione; GSSG: Oxidized glutathione; GSH:GSSG: Reduced–oxidized glutathione ratio; H_2_O_2_: Hydrogen peroxide; IsoP: Isoprostanes; MDA: Malondialdehyde; NO_2_^•^: Nitrogen oxide; NO^•^: Nitric oxide; ONOO^−^: Peroxynitrite; O_2_^•−^: Superoxide anion; O_3_: Ozone; OH^•^: Hydroxyl radical; ORAC: Oxygen Radical Antioxidant Capacity; PC: Protein Carbonyls; RO^•^: Alcoxyl radical; ROO^•^: Peroxyl radical; ROS: Reactive Oxygen Species; RNS: Reactive Nitrogen Species; Se: Selenium; SOD: Superoxide dismutase; TAC: Total Antioxidant Capacity; TAS: Total Antioxidant Status; TRAP: Total Radical-Trapping Antioxidant Parameter; Zn: Zinc; d-ROMs: Reactive Oxygen Metabolites; 4-HNE: 4-hydroxy-2-nonenal; 8-OHdG: 8-hydroxy-2′-deoxyguanosine; 8-oxo-Gua: 8-hydroxyguanine.
